# The impact of abdominal compression on outcome in patients treated with stereotactic body radiotherapy for primary lung cancer

**DOI:** 10.1093/jrr/rru028

**Published:** 2014-05-06

**Authors:** Wambaka Ange Mampuya, Yukinori Matsuo, Nami Ueki, Mitsuhiro Nakamura, Nobutaka Mukumoto, Akira Nakamura, Yusuke Iizuka, Takahiro Kishi, Takashi Mizowaki, Masahiro Hiraoka

**Affiliations:** Department of Radiation Oncology and Image-applied Therapy, Graduate School of Medicine, Kyoto University, 54 Kawahara-cho, Shogoin, Sakyo-ku, Kyoto 606–8507, Japan

**Keywords:** abdominal compression, clinical outcome, stereotactic body radiation therapy, non-small cell lung cancer

## Abstract

The aim of this study was to evaluate the impact of abdominal compression (AC) on outcome in patients treated with stereotactic body radiotherapy (SBRT) for primary lung cancer. We retrospectively reviewed data for 47 patients with histologically proven non-small cell lung cancer and lung tumour motion ≥8 mm treated with SBRT. Setup error was corrected based on bony structure. The differences in overall survival (OS), local control (LC) and disease-free survival (DFS) were evaluated to compare patients treated with AC (*n* = 22) and without AC (*n* = 25). The median follow-up was 42.6 months (range, 1.4–94.6 months). The differences in the 3-year OS, LC and DFS rate between the two groups were not statistically significant (*P* = 0.909, 0.209 and 0.639, respectively). However, the largest difference was observed in the LC rate, which was 82.5% (95% CI, 54.9–94.0%) for patients treated without AC and 65.4% (95% CI, 40.2–82.0%) for those treated with AC. After stratifying the patients into prognostic groups based on sex and T-stage, the LC difference increased in the group with an unfavourable prognosis. The present study suggests that AC might be associated with a worse LC rate after SBRT using a bony-structure-based set-up.

## INTRODUCTION

Stereotactic body radiotherapy (SBRT), a rapidly evolving technique, has proven to be an efficient and effective alternative to surgery to treat early-stage lung cancer. Indeed, compared with conventional radiotherapy, the high conformality and precise radiation delivery in SBRT facilitates safer delivery of an ablative dose to the tumour while minimizing the dose to the surrounding normal tissues, therefore achieving better local control (LC) rates (similar to those obtained with surgery) [1, 2]. Imaging and respiratory motion management, especially for a moving target such as a lung tumour, play a crucial role in achieving this high conformality.

Abdominal compression (AC) is a widely used respiratory motion management technique in patients treated with SBRT for lung cancer; its efficiency at reducing the amplitude of respiratory-induced tumour motion has been reported in many studies [3, 4]. However, some disadvantages have been reported related to the use of AC [4–6]. According to Bissonnette *et al*., AC can cause increased variation in tumour motion [6]. In a larger series of patients with a larger degree of tumour motion than the Bissonnette study, we reported larger interfractional variations in tumour motion amplitude in the group of patients treated with AC [5]. Heinzerling *et al*. reported difficulty in reproducing the compression effects of AC over the course of an SBRT treatment because of changes in the patient's anatomy, girth, and respiratory patterns during the treatment period [4]. These changes may increase the probability of target underdosing and overdosing of surrounding normal tissues. This is critical, considering the high dose per fraction used in SBRT that may affect the treatment outcome.

In this study, we retrospectively reviewed the data of patients with histologically proven non-small cell lung cancer (NSCLC) who underwent SBRT with and without AC, and sought to clarify the effect of AC on the clinical outcome.

## MATERIALS AND METHODS

### Patient characteristics

With the approval of the Institutional Review Board of Kyoto University Hospital, we reviewed the data of 98 patients with histologically proven NSCLC who had undergone SBRT in our institution between January 2004 and March 2009. The eligibility criteria for SBRT treatment in our institution have been described in previous reports [7]. Of the 98 patients, we retrospectively analysed 47 with tumour motion ≥8 mm, who were potential candidates for AC—see the treatment procedure section below. AC was used in 25 (53.2%) of 47 patients. Of the remaining 22 patients, the reasons for avoiding AC were as follows: history of abdominal surgery (*n* = 3), abdominal aortic aneurysm (*n* = 2), unexpected increase in tumour motion (*n* = 3), inability to significantly reduce the tumour motion amplitude (*n* = 10), and discomfort (*n* = 1). In three patients, the oncologist judged the AC device unnecessary. We subsequently divided the patients into two prognostic groups based on the recursive partitioning analysis (RPA), as described by Matsuo *et al*. [7]. RPA Class I with a favourable prognosis included female or T1a tumour patients, and Class II included male patients with T1b or T2a tumours. Patient characteristics are summarised in Table 1.

**Table 1.** Patient characteristics

	Without AC (*n* = 22)	With AC (*n* = 25)	*P*-value
Sex			0.550
Male	15	17	
Female	7	8	
Age (year)	77 (63–86)	77 (58–88)	0.563
T stage (UICC 7th)			0.187
T1a	11	7	
T1b	7	8	
T2a	4	10	
RPA			0.421
Class I	14	13	
Class II	8	12	
Histology			0.420
Ad	10	15	
Sq	9	9	
LC	2	0	
NOS	1	1	
Tumour diameter (mm)	21 (11–37)	25 (19–37)	0.049
Respiratory motion w/o AC (mm)	10 (8–25)	15 (10–40)	0.008
Respiratory motion with AC (mm)		10 (4–20)	0.214*

AC = abdominal compression, RPA = recursive partitioning analysis, Ad = adenocarcinoma, Sq = squamous cell carcinoma, LC = large cell carcinoma, NOS = non-small cell lung cancer, not otherwise specified. Values are shown in number or median (range).

*P-value between respiratory motion in patients treated without AC and that with AC in those treated with AC.

### Treatment protocol

**Fig. 1.**
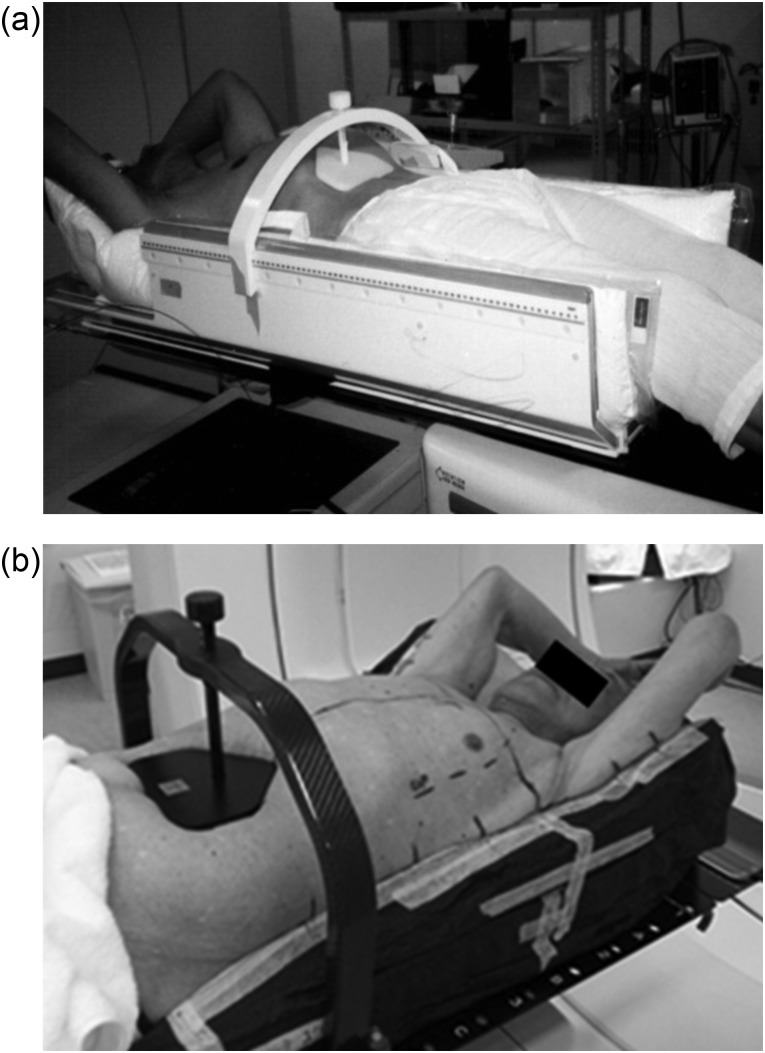
Photograph of patients immobilised with Stereotactic Body Frame (**a**) and BodyFix (**b**) with the abdomen compressed.

Up until April 2008, all patients were positioned and immobilised during simulation on a Stereotactic Body Frame (Elekta, Stockholm, Sweden), and thereafter a BodyFix vacuum cushion (Medical Intelligence, Schwabmunchen, Germany) with both arms raised was used. They subsequently underwent fluoroscopic imaging under free breathing using the Acuity Planning, Simulation, and Verification System, version 8.1 (Varian Medical Systems, Palo Alto, CA) to evaluate tumour motion. When the tumour motion observed on X-ray fluoroscopy exceeded 8 mm in the longitudinal direction, a pressure plate was used to attempt to reduce the amplitude of motion, except in patients with abdominal aneurysm or gallstones [3]. The pressure plate was placed 3–4 cm below the costal margin of the ribs below the xiphoid. The plate was connected by a graduated screw to a bar that was firmly attached to the treatment couch (BodyFix) or frame (Stereotactic Body Frame), the position of which was reproduced at each treatment. The screw was then tightened to compress the plate until the motion amplitude was decreased sufficiently. The position of the screw was recorded and reproduced during each treatment session (Fig. 1) [5]. We ensured that the compression could be tolerated over the treatment course.

The internal target volume (ITV) was delineated according to the image dataset obtained from a CT scan performed with a slow scan technique and a gantry rotation time of 4 s, or according to averaged intensity projection images derived from a four-dimensional CT scan (4DCT), depending on whether the treatment was planned before or after October 2006, respectively [8]. When the delineated ITV was found to be insufficient to encompass the respiratory motion observed on X-ray fluoroscopy, the ITVs were manually corrected according to fluoroscopic tumour motion evaluation. Planning target volumes (PTVs) were thereafter created by adding 5-mm margins to the ITVs in all directions.

The treatment plans were made using Eclipse (Varian Medical Systems) and calculated under heterogeneity correction using the Batho Power Law. They consisted of 5–8 non-coplanar, static 6-MV photon beams from the Clinac 2300C/D (Varian Medical Systems) for patients treated before April 2008, and the Novalis system (BrainLAB, Feldkirchen, Germany) thereafter. The treatment beam was collimated to the PTV with a 5-mm margin using a multileaf collimator to ensure a peripheral dose of the PTV. Set-up correction was based on bony alignment using linacgraphy for the Clinac 2300C/D and the ExacTrac X-Ray system for the Novalis system. A total dose of 48 Gy in four fractions was prescribed to the isocentre in a 1-week treatment schedule with a median overall treatment time of 5 d (range, 4–10 d).

### Evaluation

The follow-up procedure has been previously described in detail [7]. The follow-up period was defined as the period from the first day of SBRT to the last follow-up visit or date of death. Local recurrence was judged according to either histology or chest CT images showing enlargement of the tumour for at least 6 months. ^18^F-Fluorodeoxyglucose position emission tomography (FDG-PET) was recommended when local recurrence was suspected, but was not mandatory. The time of LC was defined as the duration from the beginning of SBRT treatment to the date of local recurrence.

### Statistical analyses

The chi square test and the Mann–Whitney U test were used to assess the significance of differences between the group of patients treated with and without AC.

Overall survival (OS), LC and disease-free survival (DFS) were estimated by the Kaplan–Meier method, and the differences between patient groups treated with and without AC were assessed by the log–rank test. An R version 2.13.2 with RcmdrPlugin.EZR package [9] was used for statistical analysis. Statistical significance was defined as *P* < 0.05.

## RESULTS

**Fig. 2.**
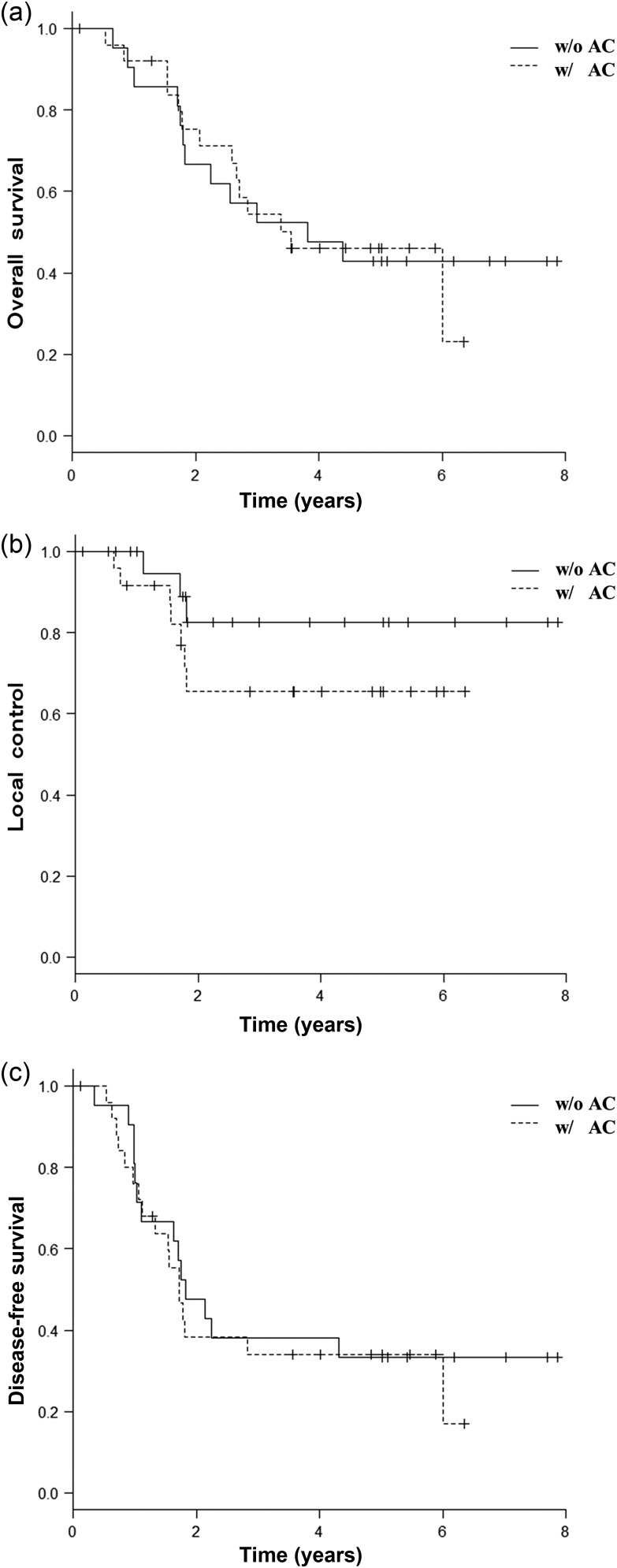
Overall survival (**a**), local control (**b**) and disease-free survival (**c**) comparing those treated with (w/AC) with those treated without abdominal compression (w/o AC) including all 47 patients.

**Fig. 3.**
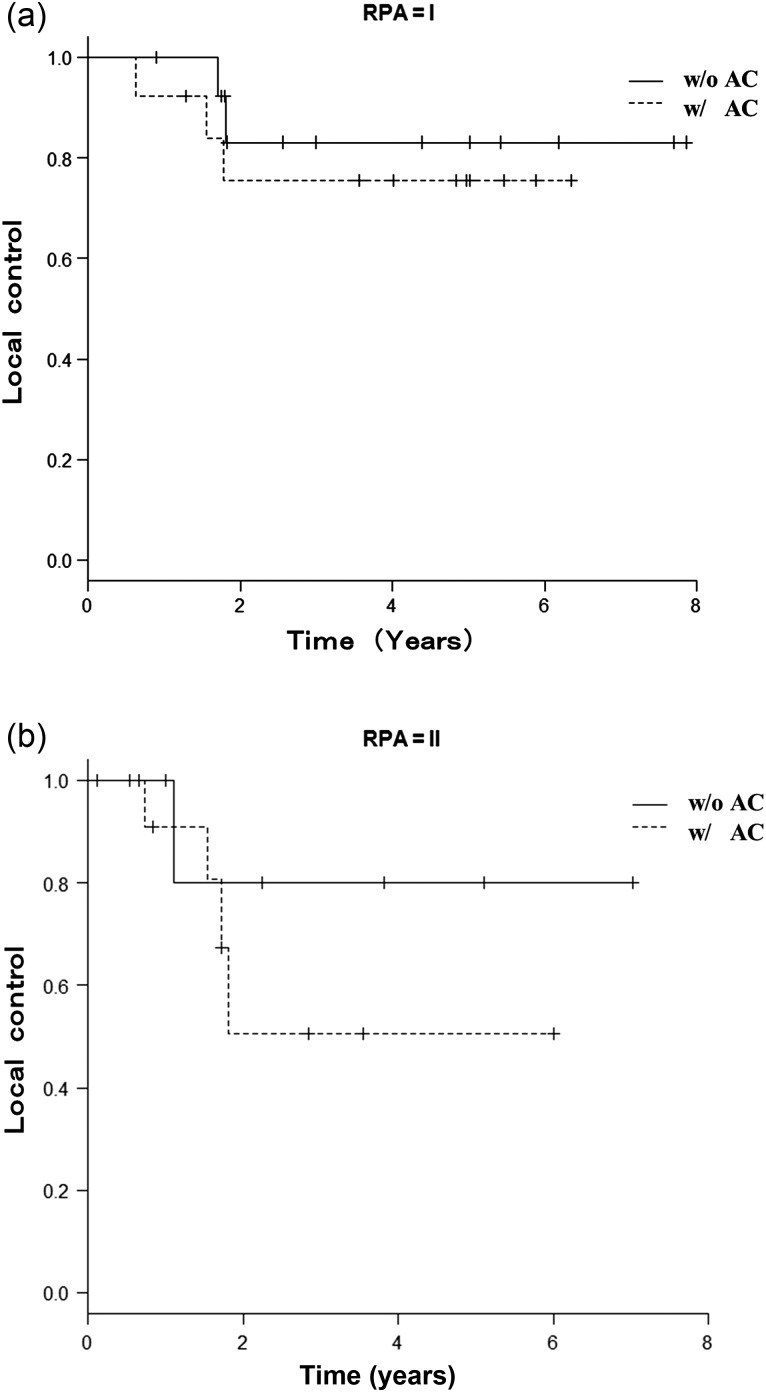
Local control in patients treated with (w/AC) and without abdominal compression (w/o AC) after stratification, according to the recursive partitioning analysis (RPA); RPA Class I (**a**) and RPA Class II (**b**).

The median follow-up period was 42.6 months (range, 1.4–94.6 months). The 3-year OS, LC and DFS rates for all 47 patients were 52.4% (95% confidence interval [CI], 29.7–70.9%), 82.5% (95% CI, 54.9–94.0%) and 38.1% (95% CI, 18.3–57.8%), respectively, for patients treated without AC and 54.4% (95% CI, 32.9–71.6%), 65.4% (95% CI, 40.2–82.0%) and 34% (95% CI, 16.3–52.6%), respectively, for patients treated with AC (Fig. 2). The differences between the two groups were not statistically significant (*P* = 0.909, 0.209 and 0.639, respectively). However, the largest difference between the two groups was observed in the LC rate. After stratification according to the RPA, 57.4% of patients were RPA Class I, and 42.6% were RPA Class II. In RPA Class I, the LC rate was 83.1% (95% CI, 47.2–95.5%) for patients treated without AC, and 75.5% (95% CI, 41.6–91.4%) for patients treated with AC (*P* = 0.527, Fig. 3a). In RPA Class II, the LC rate was 80.0% (95% CI, 20.4–96.9%) for patients treated without AC, and 50.5% (95% CI, 13.6–79.2%) for patients treated with AC (*P* = 0.394, Fig. 3b). T-stage distribution (T1a/T1b/T2a) in patients who experienced local recurrence was 1/2/0 in the group without AC, and 2/1/4 in the AC group, respectively.

## DISCUSSION

Although AC efficiently reduced the overall amplitude of tumour motion, the day-to-day reproducibility of its compression remains questionable [4]. This may result in greater interfraction variation in tumour motion and geometric miss in the dose delivery [6]. Moreover, Bouilhol *et al*. reported minor benefits or even unwanted effects, such as increased tumour motion and ITV for tumours located far from the diaphragm [10]. Considering the above disadvantages, the current study investigated the impact of the use of AC on clinical outcome. Several retrospective and prospective studies of the clinical outcomes of using SBRT to treat lung cancer have been published [11–15]. However, the present study is, to our knowledge, the first to compare the clinical outcome between patients treated with and without AC while undergoing SBRT for lung cancer.

The 3-year OS rate reported in the literature ranges from 43–60% [11–15]. Onimaru *et al*. [11], using 48 Gy in four fractions over 1 week, reported an OS rate of 53%. The present study, using the same dose fractionation schedule, demonstrated 3-year OS rates of 52.4% and 54.4% for patients treated without and with AC, respectively. The 3-year OS rate in patients treated without and with AC was consistent with recent reports; the use of AC did not affect the OS rate. We also found no difference in the 3-year DFS between the two groups.

However, our main finding was the large difference in the 3-year LC rate between patients treated with and without AC. The 3-year LC rate was 82.5% for patients treated without compression for a median follow-up period of 42.6 months. This is consistent with previous reports. However, the LC rate was 65.4% in patients treated with AC, which was lower than reported previously. To account for possible confounding factors between the group treated with AC and the one treated without, we stratified the patients according to prognostic groups based on RPA, as described by Matsuo *et al*. [7]. Patients in the RPA Class I group with a better prognosis had better LC rates than those in RPA Class II, regardless of the use of AC. In both RPA Class I and RPA Class II groups, the LC rate was higher in patients treated without than in those treated with AC, with a larger difference between the two groups in the RPA Class II patients. This suggests that RPA Class II patients may be more sensitive to the negative effects of AC, although the difference between the two groups was statistically insignificant.

We previously reported greater interfraction variation in lung tumour position in patients treated with abdominal compression in the absence of soft tissue target matching [5]. Ikushima *et al*. evaluated the changes in soft tissue tumour position during hypofractionated, in-room, CT-guided SBRT for lung cancer [16]. They reported a trend in ITV movement in any direction of more than 5 mm away from the original position from the first fraction to the last fraction in more than 20% of the patients when soft-tissue-based alignment was not used. In the present study, the treatment for all patients was based on bony alignment. Therefore, the use of AC in addition to the lack of daily soft tissue image guidance may explain the worse LC rate observed in the group of patients treated with AC. Our institution currently uses cone-beam computed tomography for tumour-based setup.

The results of the present study should, however, be interpreted with caution because of the small sample size and also due to subtle differences between the two groups, such as a larger initial degree of tumour motion in patients treated with AC. Although the motion was reduced after the use of AC, we cannot confirm that the negative impacts of larger degrees of motion were also prevented. Moreover, a greater proportion of patients with T2a tumours in the group of patients treated with AC may have possibly influenced the outcomes in this particular group.

AC efficiently reduces tumour motion, and therefore decreases the chance of toxicity. However, the effect of AC on local control has not been investigated. Considering that confounding factors could not be eliminated, the present study suggests that AC potentially worsens local control. Therefore we recommend recourse to an image-guided radiotherapy technique allowing soft tissue target matching during or immediately before treatment when AC is planned, particularly for patients with an unfavourable prognosis.

## FUNDING

This work was supported by the Japan Society for the Promotion of Science (JSPS) through the ‘Funding Program for World-Leading Innovative R&D on Science and Technology (FIRST Program)’ initiated by the Council for Science and Technology Policy (CSTP) and Grant-in-Aid for Scientific Research (A) No. 25253078. Funding to pay the Open Access publication charges for this article was provided by the Grant-in-Aid for Scientific Research (A).
